# Examination of Joint Effusion Magnetic Resonance Imaging of Patients with Temporomandibular Disorders with Disc Displacement

**DOI:** 10.3390/jimaging10100241

**Published:** 2024-09-27

**Authors:** Fumi Mizuhashi, Ichiro Ogura, Ryo Mizuhashi, Yuko Watarai, Makoto Oohashi, Tatsuhiro Suzuki, Momoka Kawana, Kotono Nagata

**Affiliations:** 1 Department of Removable Prosthodontics, The Nippon Dental University School of Life Dentistry at Niigata, Niigata 951-8580, Japan; watarai@ngt.ndu.ac.jp; 2 Functional Occlusal Treatment, The Nippon Dental University Graduate School of Life Dentistry at Niigata, Niigata 951-8580, Japan; tatsu@ngt.ndu.ac.jp (T.S.); kawana.momo@ngt.ndu.ac.jp (M.K.); nagatako35@ngt.ndu.ac.jp (K.N.); 3 Department of Oral and Maxillofacial Radiology, The Nippon Dental University School of Life Dentistry at Niigata, Niigata 951-8580, Japan; ogura@ngt.ndu.ac.jp; 4 Comprehensive Dental Care, The Nippon Dental University Niigata Hospital, Niigata 951-8580, Japan; ryo-mz@ngt.ndu.ac.jp; 5 Department of Dental Anesthesia and General Health Management, The Nippon Dental University School of Life Dentistry at Niigata, Niigata 951-8580, Japan; oohashi@ngt.ndu.ac.jp

**Keywords:** magnetic resonance imaging, temporomandibular disorders with disc displacement, joint effusion

## Abstract

In this study, we investigated joint effusion in patients with temporomandibular disorders (TMDs) with disc displacement. The magnetic resonance (MR) images of 97 temporomandibular joints (TMJs) were evaluated, and the appearance of joint effusion was investigated. Myofascial pain and TMJ pain were considered in addition to the duration from manifestation. Disc displacement with and without reduction, as well as the region and the area of joint effusion, were investigated using the MR images. Fisher’s test was used for the analyses. Joint effusion was recognized in 70 TMJs, including 55 in the superior articular cavity, 1 in the inferior articular cavity, and 14 in both the superior and inferior articular cavities. The appearance of joint effusion did not differ with the existence of myofascial pain or TMJ pain. The region of joint effusion did not differ between disc displacement with and without reduction. A larger area of joint effusion was recognized in disc displacement without reduction (*p* < 0.05). The results showed that the amount of synovial fluid in the joint effusion did not change with the existence of myofascial pain or TMJ pain. Joint effusion commonly appeared in disc displacement without reduction.

## 1. Introduction

Temporomandibular disorders (TMDs) involve many clinical problems of the masticatory muscle, the temporomandibular joint (TMJ), and surrounding structures [1]. A TMD is the second most commonly occurring musculoskeletal condition that causes a decline in the quality of life of patients [2]. Pain, joint noises, and disturbances in jaw opening [3,4] are the most frequent signs and symptoms of a TMD [5]. Patients with a TMD often describe a concomitant appearance of headaches [6,7] and sleep disturbances. A TMD is classified as myofascial pain, arthralgia, disc displacement with reduction, disc displacement without reduction, osteoarthrosis, etc. [1,8,9]. The prevalence of TMDs is approximately 5–12% in the adult population [10], with a reported prevalence of 34% in children with primary dentition [11]. Approximately 33% of the population has TMD symptoms, and 3.6–7.0% of the population has sufficiently severe TMD symptoms that require treatment [12]. There is a peak occurrence between 20 and 40 years of age [13,14,15], and it is more common in women [16]. The most common form of TMJ dysfunction is disc displacement, and the prevalence of disc displacement has been reported to be 77–89% [17,18], 48.9% [19], or 41% [20]. The frequency of symptoms differs between reports. Displaced discs in TMJs lose their normal disc–condyle relationship in the closed-mouth position, and the disc can be displaced to the front, inner area, outer area, or posterior of the condyle [21]. Most articular discs show anterior displacement of the disc [8,9], and disc displacement causes internal derangement [5]. Anterior disc displacement is an intracapsular dysfunction, with degenerative changes in the disc of the TMJ [22] that cause symptoms such as TMJ pain, joint noises, and disturbances in jaw opening. The most common type of disc displacement is disc displacement with reduction [20]. Disc displacement without reduction is recognized in approximately one-quarter to one-third of symptomatic patients [23,24] and may be a risk factor for osteoarthrosis [25,26] or adhesion [27].

The detection of disc displacement is a prerequisite for the diagnosis of a TMD and for decision-making regarding treatment plans. Magnetic resonance imaging (MRI) is suitable for the diagnosis of disc displacement [28] and for the diagnosis of a TMD [29,30]. MRI provides the best soft-tissue visualization of the TMJ, with notable contrast resolution and spatial resolution in an ionizing radiation-free manner [31]. In one study, the interobserver agreement of the position of the disc was demonstrated to be 95%, and the percentage of osseous changes was 97% [32]. MRI is the established diagnostic method for TMDs; the change in the position of the TMJ disc before and after treatment can be used as one of the indicators to evaluate the treatment outcome. Conventionally, MRI is commonly performed using 1.5 T or 3.0 T scanners, with a head coil or a TMJ surface coil for the detection of TMDs [33]. A clinical examination often includes static T1-emphasized images, T2-emphasized images, and proton-density-emphasized images in both the closed-mouth and maximum opened-mouth positions [34]. The shape of a normal TMJ disc is biconcave, presenting a less than normal intensity on an MR image. A disc displacement is diagnosed using proton density with a high contrast in MRI, and joint effusion is detected using a T2-emphasized image. Joint effusion is a condition where the retention of the synovial fluid is observed as a hyperintense area in T2-emphasized images, and this may reflect the state of inflammation in the TMJ [35]. There is no consensus on the interpretation of joint effusion: one study indicated that joint effusion was related to disc displacement and arthralgia [35], but another showed that joint effusion was observed in TMJs without inflammation [36]. TMDs showing joint effusion are common in MRI, but it is difficult to decide the condition of joint effusion in MR images or the status of TMJs in patients with TMDs. Therefore, the factors leading to joint effusion at a clinical site must be elucidated. In this study, we examined the condition of disc displacement with and without reduction and elucidated the factors relating to joint effusion in patients with TMDs with disc displacement.

## 2. Materials and Methods

The subjects of this study were 97 TMJs of the TMD patients (12 men and 85 women; mean age: 43.6 ± 19.3 years) who attended the Temporomandibular Joint Disorders & Bruxism Clinic of the Nippon Dental University Niigata Hospital. All patients were diagnosed with TMDs and anterior disc displacement (with or without reduction) by means of MRI. Interviews (to ascertain myofascial pain, TMJ pain, and duration from manifestation), clinical presentations (myofascial pain and TMJ pain), and medical examinations were performed before MRI. The characteristics of the subjects in this study were identified as myofascial pain (with pain: *n* = 53; without pain: *n* = 44), TMJ pain (with pain: *n* = 44; without pain: *n* = 53), and disturbances in mouth opening (with disturbance: *n* = 48; without disturbance: *n* = 49). From MRI, 35 TMJs were diagnosed as anterior disc displacement with reduction, and 62 TMJs were diagnosed as anterior disc displacement without reduction. This retrospective study was approved by the ethics committee of our institution.

The MRI equipment used in this study was a 1.5 Tesla MR unit (EXCELART VantageMRT-2003; Canon Medical Systems, Otawara, Japan) with a surface coil for the TMJ. MRI included proton-density-weighted sagittal and coronal imaging. The two positions of the mouth were set as a closed mouth and the maximum-opened mouth (repetition time/echo time: 2000 ms/18 ms; field of view: 130 mm × 130 mm; matrix size: 256 × 224; 1 acquisition). T2-weighted sagittal and coronal imaging was also included using two positions of the mouth. These were a closed mouth and the maximum-opened mouth (repetition time/echo time: 3500 ms/100 ms; field of view: 130 mm × 130 mm; matrix size: 256 × 192; 2 acquisitions) [37,38]. The images were independently evaluated by two radiologists with clinical experience of more than ten years. Any discrepancies were resolved via consensus. The positions of the TMJ disc were confirmed first. Normal positioning of the TMJ disc is between the mandibular condyle and fossa. The positioning of the TMJ disc in this study showed anterior disc displacement of various shapes in the proton-density-weighted sagittal imaging. The detection of joint effusion was investigated using T2-emphasized MRI. Joint effusion was identified as hyperintense areas in the superior or inferior articular cavities from the T2-emphasized image. In this study, the region of joint effusion was classified as the superior articular cavity, the inferior articular cavity, and both the superior and inferior articular cavities. The amount of synovial fluid in the joint effusion was also investigated using T2-emphasized images and classified as Grade 0 (no fluid), Grade 1 (with punctiform or filamentous fluid), Grade 2 (cingulate fluid), or Grade 3 (plenitude fluid).

In this study, we examined the condition of TMD patients with disc displacement with and without reduction in relation to myofascial pain, TMJ pain, and joint effusion. The appearance of myofascial pain and TMJ pain with disc displacement with and without reduction was compared. The region of joint effusion and the amount of synovial fluid in the joint effusion were investigated for disc displacement with and without reduction. The factors relating to joint effusion in TMD patients with disc displacement with and without reduction were examined. The site of joint effusion was confirmed first, and then the relationship between the duration from manifestation and the region of joint effusion or the amount of synovial fluid in the joint effusion was investigated. The region of joint effusion and the amount of synovial fluid in the joint effusion were also investigated to ascertain their relationship to myofascial pain or TMJ pain.

Statistical analyses were performed using Fisher’s test. The differences in the appearance of myofascial pain or TMJ pain for disc displacement with and without reduction were compared using Fisher’s test, as were the differences in the region of joint effusion and the amount of synovial fluid in the joint effusion. The differences in the region of joint effusion and the amount of synovial fluid in the joint effusion along with the existence of myofascial pain or TMJ pain were also analyzed using Fisher’s test. Statistical analyses were performed using statistical analysis software (SPSS 17.0, SPSS JAPAN, Tokyo, Japan). Differences with α < 0.05 were considered to be significant.

## 3. Results

**Figure 1:**
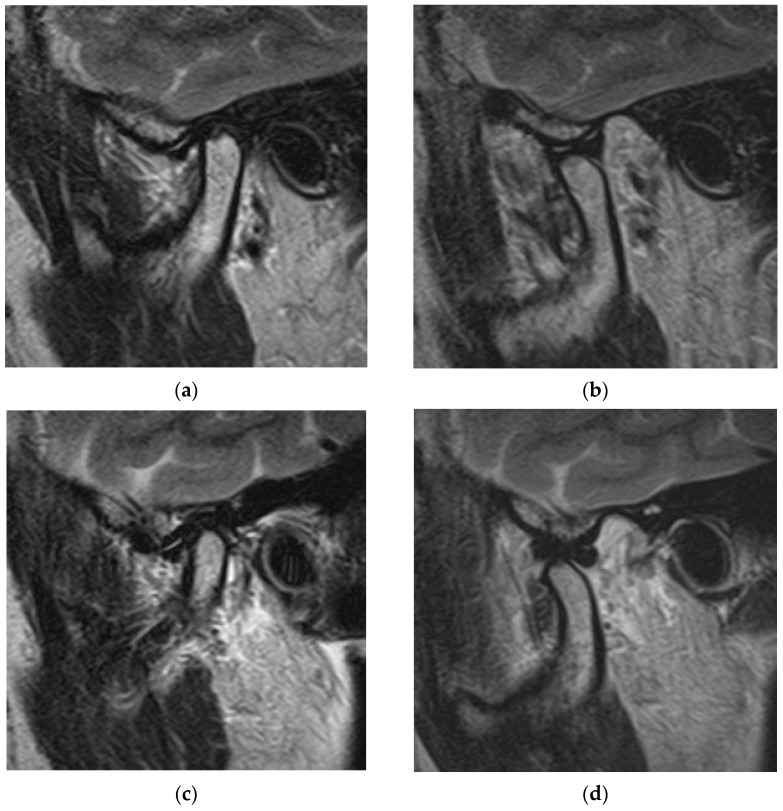
MR image of a 56-year-old woman with TMDs of right and left disc displacement with reduction. (**a**) Right TMJ: T2-weighted sagittal oblique cross-section imaging in the closed-mouth position; (**b**) right TMJ: T2-weighted sagittal oblique cross-section imaging in the opened-mouth position; (**c**) left TMJ: T2-weighted sagittal oblique cross-section imaging in the closed-mouth position; (**d**) left TMJ: T2-weighted sagittal oblique cross-section imaging in the opened-mouth position.

Figure 1 shows an MR image of a 56-year-old woman with disc displacement with reduction. T2-weighted sagittal oblique cross-section imaging of the right TMJ revealed an anterior disc displacement in the closed-mouth position (Figure 1a) and a reduction in the disc in the opened-mouth position (Figure 1b). T2-weighted sagittal oblique cross-section imaging of the left TMJ revealed anterior disc displacement in the closed-mouth position (Figure 1c) and a reduction in the disc in the opened-mouth position (Figure 1d).

**Figure 2:**
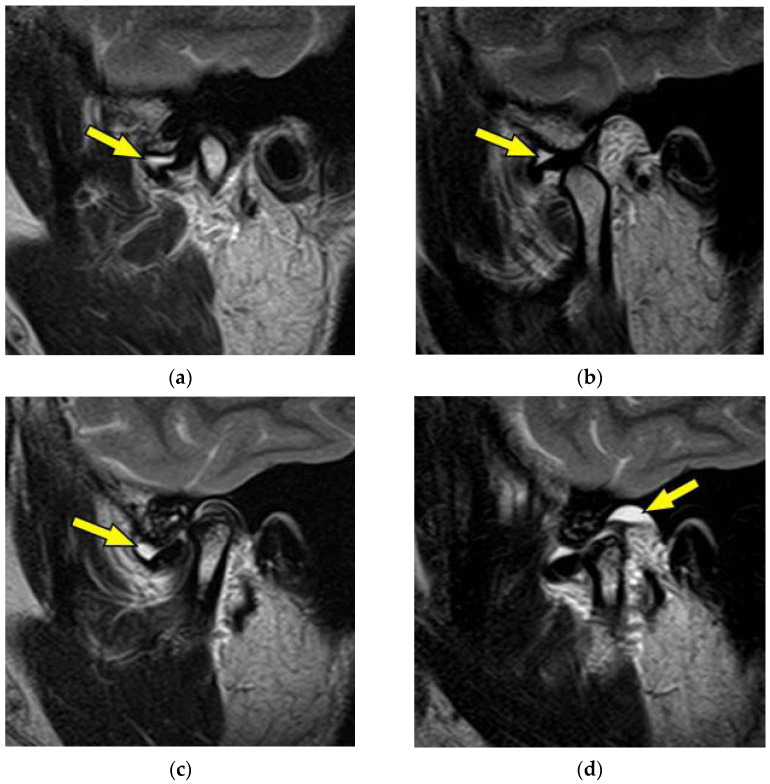
MR image of a 38-year-old woman with TMDs of right and left disc displacement without reduction. (**a**) Right TMJ: T2-weighted sagittal oblique cross-section imaging in the closed-mouth position; (**b**) right TMJ: T2-weighted sagittal oblique cross-section imaging in the opened-mouth position; (**c**) left TMJ: T2-weighted sagittal oblique cross-section imaging in the closed-mouth position; (**d**) left TMJ: T2-weighted sagittal oblique cross-section imaging in the opened-mouth position. Arrows indicate the region of joint effusion.

Figure 2 shows an MR image of a 38-year-old woman with disc displacement without reduction. T2-weighted sagittal oblique cross-section imaging of the right TMJ showed an anterior disc displacement in the closed-mouth position (Figure 2a). The disc without reduction in the opened-mouth position is shown in Figure 2b. Joint effusion was recognized in the superior articular cavity of the right TMJ (yellow arrow). T2-weighted sagittal oblique cross-section imaging of the left TMJ showed an anterior disc displacement in the closed-mouth position (Figure 2c). The disc without reduction in the opened-mouth position is shown in Figure 2d. Joint effusion was recognized in the superior articular cavity of the left TMJ (yellow arrow).

The appearance of myofascial pain did not differ between disc displacement with and without reduction (*p* = 0.208; Table 1). The appearance of TMJ pain differed between disc displacement with reduction and disc displacement without reduction (*p* < 0.01). TMJ pain was commonly present with disc displacement without reduction (Table 1).

Of the 97 TMJs, 27 did not present joint effusion. Joint effusion appeared in the superior articular cavity (*n* = 55), the inferior articular cavity (*n* = 1), or both the superior and inferior articular cavities (*n* = 14). The most common site for joint effusion was the superior articular cavity.

The region of joint effusion was investigated for disc displacement with and without reduction. The results showed that there were no statistically significant differences in the region of joint effusion between disc displacement with and without reduction (*p* = 0.825, Table 2).

The amount of joint-effusion synovial fluid differed between disc displacement with reduction and disc displacement without reduction. The amount of synovial fluid classified as Grade 2 (cingulate) was larger for disc displacement with reduction, and that of Grade 3 (plenitude) was larger for disc displacement without reduction (*p* = 0.024; Table 3).

The relationship between the duration from manifestation and the region of joint effusion was investigated. The duration from manifestation tended to differ in relation to the region of joint effusion. The duration was longest when joint effusion was observed in the inferior articular cavity only (1080 days from manifestation) compared with no appearance (143 days), the superior articular cavity only (369 days), and both the superior and inferior articular cavities (213 days). The duration from manifestation also differed in relation to the amount of synovial fluid in the joint effusion. The duration was shorter when plenitude fluid was observed (Grade 3; 143 days) or when no synovial fluid was observed (Grade 0; 143 days). The duration from manifestation was longer when punctiform or filamentous fluid was observed (Grade 1; 312 days) or when cingulate fluid was observed (Grade 2; 428 days).

The region of joint effusion was investigated in relation to myofascial pain. The results showed that there were no significant differences in the region of joint effusion by the existence of myofascial pain (*p* = 0.956; Table 4). The region of joint effusion was also examined in relation to TMJ pain. The results also showed no significant differences in the region of joint effusion by the existence of TMJ pain (*p* = 0.336; Table 4).

The amount of synovial fluid in the joint effusion was examined in relation to myofascial pain. The results showed that there was no statistically significant difference in the amount of synovial fluid in the joint effusion by the existence of myofascial pain (*p* = 0.570; Table 5). The amount of synovial fluid in the joint effusion was also examined in relation to TMJ pain. The results also showed no statistically significant difference in the amount of synovial fluid in the joint effusion by the existence of TMJ pain (*p* = 0.307; Table 5).

## 4. Discussion

In this study, we investigated the conditions of the most common form of TMJ dysfunction—disc displacements with and without reduction—in relation to the appearance of joint effusion identified using MRI. The region of joint effusion and the amount of joint-effusion synovial fluid were investigated because the state of joint effusion has not yet been elucidated.

An analysis of the relationship between pain and the condition of the disc displacement revealed that myofascial pain did not differ between disc displacement with and without reduction, but TMJ pain commonly appeared in disc displacement without reduction. The TMJ is composed of the articular condyle, articular fossa, articular disc, fibrous capsule, and synovial membrane [39]. A disc displacement without reduction is a condition where the articular disc loses the normal disc–condyle relationship, both in closed-mouth and opened-mouth positions. The articular disc works as cushioning between the articular condyle and the articular fossa. If the disc is displaced, there is no cushion between the articular condyle and the articular fossa; thus, internal derangement easily occurs [5]. The load on the TMJ is larger for disc displacement without reduction compared with that of disc displacement with reduction; thus, TMJ pain commonly appears with disc displacements without reduction.

Joint effusion most frequently appeared in the superior articular cavity; appearances in the inferior articular cavity were rare. This tendency was the same for disc displacement with and without reductions. This indicates that synovial fluid is frequently stored in the superior articular cavity. The superior articular cavity is stimulated by mandible movement; that stimulation may result in the storing of synovial fluid in the superior articular cavity. The amount of joint-effusion synovial fluid was larger in disc displacement without reduction compared with disc displacement with reduction. There was consistently no cushion between the articular condyle and the articular fossa in the TMJ of disc displacement without reduction. The load on the TMJ was larger for TMJs with disc displacement without reduction compared with those of disc displacement with reduction. Therefore, we ascertained that synovial fluid was mainly stored under the condition of disc displacement without reduction.

The appearance of joint effusion tended to differ according to the duration from manifestation; joint effusion tended to appear in the inferior articular cavity when the duration from manifestation was longer. The amount of joint-effusion synovial fluid was larger when the duration from manifestation was shorter, and the amount of synovial fluid decreased when the duration from manifestation was longer. In a clinical situation, a change in the appearance of joint effusion in TMD patients is often observed, and joint effusion tends to disappear as the day progresses. It has been suggested that joint effusion appears during the early stage of TMDs and reflects the condition of inflammation on the TMJ [35]. Further research should be undertaken to establish the conditions of joint effusion by investigating changes in the joint-effusion appearance over time.

The region of joint effusion did not differ with the existence of myofascial pain or TMJ pain. The amount of joint-effusion synovial fluid did not change with the existence of myofascial pain or TMJ pain. These results suggest that joint effusion observed using MRI did not reflect the acute condition of the TMJ. A previous report indicated that joint effusion is related to arthralgia [35]. In this study, approximately 80% of TMD patients with TMJ pain presented with joint effusion, but 20% did not, despite the identification of TMJ pain. These results supported the observations from another report, where joint effusion was observed even for TMJs without inflammation [36]. Joint effusion was observed in approximately 66% of TMD patients without TMJ pain in this study, indicating that myofascial pain or TMJ pain does not influence the appearance of joint effusion in TMD patients with disc displacement with and without reduction.

In this study, we investigated the factors relating to joint effusion in TMD patients with disc displacement. The results suggest that joint effusion appears during the early period from the manifestation of TMDs. The amount of joint-effusion synovial fluid was larger for disc displacement without reduction compared with disc displacement with reduction. Joint effusion identified using MRI could be one of the parameters used to indicate the condition of TMD patients. Joint effusion appears when the load on the TMJ increases. Changes in joint effusion of patients over time should be investigated in future research studies to ascertain the alterations to joint effusion. If the mechanism of joint effusion is identified, the conditions of TMJs could be revealed by MRI, and diagnostic imaging using MRI could be used to prepare treatment plans.

The results of this study suggest that TMJ pain and joint effusion commonly appear in patients with disc displacement without reduction. The amount of synovial fluid was larger in the patients with disc displacement without reduction. These results suggest that disc displacement without reduction is a more severe condition than disc displacement with reduction. We recommend that care is taken not to add excessive loads.

## 5. Conclusions

In this study, we investigated the TMJs of subjects with disc displacement with and without reduction using MRI. The results revealed that the amount of joint-effusion synovial fluid did not change with the existence of myofascial pain or TMJ pain. Joint effusion commonly appeared in patients with disc displacement without reduction. A larger amount of synovial fluid was recognized in disc displacement without reduction. This knowledge is important when determining the condition of TMJs using MRI.

## Figures and Tables

**Table 1:** Appearance of myofascial pain and temporomandibular joint pain in TMD patients with disc displacements with and without reduction.

Factor	Disc Displacement with Reduction	Disc Displacement without Reduction	Total	*p* Value
Myofascial pain	35 (36.1%)	62 (63.9%)	97 (100%)	0.208
With pain	16 (30.2%)	37 (69.8%)	53 (100%)	
Without pain	19 (43.2%)	25 (56.8%)	44 (100%)	
Temporomandibular joint pain	35 (36.1%)	62 (63.9%)	97 (100%)	0.005 **
With pain	9 (20.5%)	35 (79.5%)	44 (100%)	
Without pain	26 (49.1%)	27 (50.9%)	53 (100%)	

** *p* < 0.01.

**Table 2:** Region of joint effusion for TMD patients with disc displacement with and without reduction.

Factor	No Appearance	Superior Articular Cavity	Inferior Articular Cavity	Both Superior and Inferior Articular Cavities	*p* Value
Disc displacement with reduction	9 (33.3%)	22 (40.0%)	0 (0%)	4 (28.6%)	0.825
Disc displacement without reduction	18 (66.7%)	33 (60.0%)	1 (100%)	10 (71.4%)	
Total	27 (100%)	55 (100%)	1 (100%)	14 (100%)	

**Table 3:** Amount of joint-effusion synovial fluid in TMD patients with disc displacement with and without reduction.

Factor	Grade 0 (No Fluid)	Grade 1 (Fluid with Punctiform or Filamentous)	Grade 2 (Fluid with Cingulate)	Grade 3 (Fluid with Plenitude)	*p* Value
Disc displacement with reduction	9 (33.3%)	4 (36.4%)	22 (46.8%)	3 (12.0%)	0.024 *
Disc displacement without reduction	18 (66.7%)	7 (63.6%)	25 (53.2%)	22 (88.0%)	
Total	27 (100%)	11 (100%)	47 (100%)	25 (100%)	

* *p* < 0.05.

**Table 4:** Region of joint effusion and the appearance of myofascial pain and TMJ pain.

Factor	No Appearance	Superior Articular Cavity	Inferior Articular Cavity	Both Superior and Inferior Articular Cavities	*p* Value
Myofascial pain	27 (100%)	55 (100%)	1 (100%)	14 (100%)	0.956
With pain	15 (55.6%)	30 (54.5%)	1 (100%)	7 (50.0%)	
Without pain	12 (44.4%)	25 (45.5%)	0 (0%)	7 (50.0%)	
Temporomandibular joint pain	27 (100%)	55 (100%)	1 (100%)	14 (100%)	0.336
With pain	9 (33.3%)	28 (50.9%)	0 (0%)	7 (50.0%)	
Without pain	18 (66.7%)	27 (49.1%)	1 (100%)	7 (50.0%)	

**Table 5:** Amount of joint-effusion synovial fluid and the appearance of myofascial pain and TMJ pain.

Factor	Grade 0 (No Fluid)	Grade 1 (with Punctiform or Filamentous Fluid)	Grade 2 (Cingulate Fluid)	Grade 3 (Plenitude Fluid)	*p* Value
Myofascial pain	27 (100%)	10 (100%)	39 (100%)	21 (100%)	0.570
With pain	15 (55.6%)	6 (60.0%)	23 (59.0%)	9 (42.9%)	
Without pain	12 (44.4%)	4 (40.0%)	16 (41.0%)	12 (57.1%)	
Temporomandibular joint pain	27 (100%)	10 (100%)	39 (100%)	21 (100%)	0.307
With pain	9 (33.3%)	6 (60.0%)	17 (43.6%)	12 (57.1%)	
Without pain	18 (66.7%)	4 (40.0%)	22 (56.4%)	9 (42.9%)	

## Data Availability

The data presented in this study are available on request from the corresponding author.

## References

[1] Wadhwa S., Kapila S. (2008). TMJ Disorders: Future innovations in diagnostics and therapeutics. J. Dent. Educ..

[2] Almeida F.T., Pacheco-Pereira C., Flores-Mir C., Le L.H., Jaremko J.L., Major P.W. (2019). Diagnostic ultrasound assessment of temporomandibular joints: A systematic review and meta-analysis. Dentomaxillofac. Radiol..

[3] Salamon N.M., Casselman J.W. (2020). Temporomandibular joint disorders: A pictorial review. Semin. Musculoskelet. Radiol..

[4] Kalladka M., Young A., Thomas D., Heir G.M., Quek S.Y.P., Khan J. (2022). The relation of temporomandibular disorders and dental occlusion: A narrative review. Quintessence Int..

[5] Herb K., Cho S., Stiles M.A. (2006). Temporomandibular joint pain and dysfunction. Curr. Pain Headache Rep..

[6] Bohm P.E., Stancampiano F.F., Rozen T.D. (2018). Migraine headache: Updates and future developments. Mayo Clin. Proc..

[7] Gonçalves D.A., Bigal M.E., Jales L.C., Camparis C.M., Speciali J.G. (2010). Headache and symptoms of temporomandibular disorder: An epidemiological study. Headache.

[8] Manfredini D., Piccotti F., Ferronato G., Guarda-Nardini L. (2010). Age peaks of different RDC/TMD diagnoses in a patient population. J. Dent..

[9] Gauer R.L., Semidey M.J. (2015). Diagnosis and treatment of temporomandibular disorders. Am. Fam. Physician.

[10] Tresoldi M., Dias R., Bracci A., Segù M., Guarda-Nardini L., Manfredini D. (2021). Magnetic resonance imaging evaluation of closed-mouth TMJ disc-condyle relationship in a population of patients seeking for temporomandibular disorders advice. Pain Res. Manag..

[11] Bonjardim L.R., Gaviao M.B., Carmagnani F.G., Pereira L.J., Castelo P.M. (2003). Signs and symptoms of temporomandibular joint dysfunction in children with primary dentition. J. Clin. Pediatr. Dent..

[12] Ouanounou A., Goldberg M., Haas D.A. (2017). Pharmacotherapy in temporomandibular disorders: A review. J. Can. Dent. Assoc..

[13] Manfredin D., Guarda-Nardini L., Winocur E., Piccotti F., Ahlberg J., Lobbezoo F. (2011). Research diagnostic criteria for temporomandibular disorders: A systematic review of axis I epidemiologic findings. Oral Surg. Oral Med. Oral Pathol. Oral Radiol. Endodontol..

[14] Loster J.E., Osiewicz M.A., Groch M., Ryniewicz W., Wieczorek A. (2017). The prevalence of TMD in Polish young adults. J. Prosthodont..

[15] Calixtre L.B., Grüninger B.L., Chaves T.C., Oliveira A.B. (2014). Is there an association between anxiety/depression and temporomandibular disorders in college students?. J. Appl. Oral Sci..

[16] Nilsson I.M., Drangsholt M., List T. (2009). Impact of temporomandibular disorder pain in adolescents: Differences by age and gender. J. Orofac. Pain.

[17] Lai L., Huang C., Zhou F., Xia F., Xiong G. (2020). Finite elements analysis of the temporomandibular joint disc in patients with intra-articular disorders. BMC Oral Health.

[18] Tasaki M.M., Westesson P.L., Isberg A.M., Ren Y.F., Tallents R.H. (1996). Classification and prevalence of temporomandibular joint disk displacement in patients and symptom-free volunteers. Am. J. Orthod. Dentofac. Orthop..

[19] Osiewicz M.A., Lobbezoo F., Loster B.W., Loster J.E., Manfredini D. (2018). Frequency of temporomandibular disorders diagnoses based on RDC/TMD in a Polish patient population. Cranio.

[20] Miernik M., Więckiewicz W. (2015). The basic conservative treatment of temporomandibular joint anterior disc displacement without reduction—Review. Adv. Clin. Exp. Med..

[21] Huang D., Liu L., Zhai X., Wang Y., Hu Y., Xu X., Li H., Jiang H. (2023). Association between chewing side preference and MRI characteristics in patients with anterior disc displacement of the temporomandibular joint. J. Stomatol. Oral Maxillofac. Surg..

[22] Taskaya-Yilmaz N., Ogutcen-Toller M. (2001). Magnetic resonance imaging evaluation of temporomandibular joint disc deformities in relation to type of disc displacement. J. Oral Maxillofac. Surg..

[23] Larheim T.A., Westesson P.L., Sano T. (2001). Temporomandibular joint disk displacement: Comparison in asymptomatic volunteers and patients. Radiology.

[24] Giraudeau A., Cheynet F., Mantout B., Philip E., Orthlieb J.D. (2008). Prevalence and distribution of intracapsular derangement of TMJ in an asymptomatic and a symptomatic population. Int. J. Stomatol. Occlusion Med..

[25] Dias I.M., Coelho P.R., Picorelli Assis N.M., Pereira Leite F.P., Devito K.L. (2012). Evaluation of the correlation between disc displacements and degenerative bone changes of the temporomandibular joint by means of magnetic resonance images. Int. J. Oral Maxillofac. Surg..

[26] Koh K.J., List T., Petersson A., Rohlin M. (2009). Relationship between clinical and magnetic resonance imaging diagnoses and findings in degenerative and inflammatory temporomandibular joint diseases: A systematic literature review. J. Orofac. Pain.

[27] Zhang S.Y., Liu X.M., Yang C., Cai X.Y., Chen M.J., Haddad M.S., Yun B., Chen Z. (2009). Intra-articular adhesions of the temporomandibular joint: Relation between arthroscopic findings and clinical symptoms. BMC Musculoskelet. Disord..

[28] Tomas X., Pomes J., Berenguer J., Quinto L., Nicolau C., Mercader J.M., Castro V. (2006). MR imaging of temporomandibular joint dysfunction: A pictorial review. Radiographics.

[29] Styles C., Whyte A. (2002). MRI in the assessment of internal derangement and pain within the temporomandibular joint: A pictorial essay. Br. J. Oral Maxillofac. Surg..

[30] Petersson A. (2010). What you can and cannot see in TMJ imaging—An overview related to the RDC/TMD diagnostic system. J. Oral Rehabil..

[31] Yılmaz D., Kamburoğlu K. (2019). Comparison of the effectiveness of high resolution ultrasound with MRI in patients with temporomandibular joint disorders. Dentomaxillofac. Radiol..

[32] Tasaki M.M., Westesson P.L., Raubertas R.F. (1993). Observer variation in interpretation of magnetic resonance images of the temporomandibular joint. Oral Surg. Oral Med. Oral Pathol..

[33] Manoliu A., Spinner G., Wyss M., Erni S., Ettlin D.A., Nanz D., Ulbrich E.J., Gallo L.M., Andreisek G. (2016). Quantitative and qualitative comparison of MR imaging of the temporomandibular joint at 1.5 and 3.0 T using an optimized high-resolution protocol. Dentomaxillofac. Radiol..

[34] Tomura N., Otani T., Narita K., Sakuma I., Takahashi S., Watarai J., Ohnuki T. (2007). Visualization of anterior disc displacement in temporomandibular disorders on contrast-enhanced magnetic resonance imaging: Comparison with T2-weighted, proton density-weighted, and precontrast T1-weighted imaging. Oral Surg. Oral Med. Oral Pathol. Oral Radiol. Endodontol..

[35] Petscavage-Thomas J.M., Walker E.A. (2014). Unlocking the jaw: Advanced imaging of the temporomandibular joint. AJR Am. J. Roentgenol..

[36] Ohkubo M., Sano T., Otonari-Yamamoto M., Hayakawa Y., Okano T., Sakurai K., Sato T., Sugiyama T., Ishida R. (2009). Magnetic resonance signal intensity from retrodiscal tissue related to joint effusion status and disc displacement in elderly patients with temporomandibular joint disorders. Bull. Tokyo Dent. Coll..

[37] DaSilva A.F., Shaefer J., Keith D.A. (2003). The temporomandibular joint: Clinical and surgical aspects. Neuroimaging Clin. N. Am..

[38] Toshima H., Ogura I. (2020). Characteristics of patients with TMJ osteoarthrosis on magnetic resonance imaging. J. Med. Imaging Radiat. Oncol..

[39] Okeson J.P. (2007). Joint intracapsular disorders: Diagnostic and nonsurgical management considerations. Dent. Clin. N. Am..

